# Real‐time fetal brain and placental T2* mapping at 0.55T MRI

**DOI:** 10.1002/mrm.30497

**Published:** 2025-03-10

**Authors:** Jordina Aviles Verdera, Sara Neves Silva, Kelly M. Payette, Raphael Tomi‐Tricot, Megan Hall, Lisa Story, Shaihan J. Malik, Joseph V. Hajnal, Mary A. Rutherford, Jana Hutter

**Affiliations:** ^1^ Early Life Imaging Department School of Biomedical Engineering and Imaging Sciences, King's College London London UK; ^2^ Imaging Physics and Engineering Department School of Biomedical Engineering and Imaging Sciences, King's College London London UK; ^3^ Center for MR‐Research University Children's Hospital Zurich Zurich Switzerland; ^4^ MR Research Collaborations Siemens Healthcare Limited Camberley UK; ^5^ Women's Health GSTT London UK; ^6^ Smart Imaging Lab Radiological Institute, University Hospital Erlangen Erlangen Germany

**Keywords:** fetal MRI, low cost, low‐field, relaxometry

## Abstract

**Purpose:**

To provide real‐time, organ‐specific quantitative information — specifically placental and fetal brain T2 * — to optimize and personalize fetal MRI examinations.

**Methods:**

A low‐latency setup enables real‐time processing, including segmentation, T2* fitting, and centile calculation. Two nnU‐Nets were trained on 2 989 fetal brains, and 540 placental datasets for automatic segmentation. Normative T2* curves over gestation were derived from 88 healthy cases. Prospective testing included 50 fetal MRI scans: A validation cohort (10 exams with three intra‐scan repetitions) and an evaluation cohort (40 participants). Validation was performed with Bland‐Altman assessments and Dice coefficients between repetitions, manual/automatic segmentations, and online/offline quantification.

**Results:**

T2* maps and centiles for the fetal brain and placenta were available in under one minute for all cases. The validation cohort showed robust reproducibility, with intra‐scan mean T2* differences of 1.04, −3.17, and 5.07 ms for the fetal brain and −3.15, 4.74, and 2.45 ms for the placenta. Mean T2* differences between online and offline processing were 1.63 ms and 0.16 ms for the fetal brain and placenta, respectively. Dice coefficients were 0.84±0.02 for the placenta and 0.96±0.01 for the fetal brain.

**Conclusions:**

Real‐time quantitative imaging supports personalized MR exams, optimizing sequence selection and working towards reducing recall rates. The ability to assess T2*, a potential biomarker for pregnancy complications, in real‐time opens new clinical possibilities. Future research will apply this pipeline to pregnancies affected by preeclampsia and growth restriction and explore MR‐guided fetal interventions.

## 1 INTRODUCTION

Fetal Magnetic Resonance Imaging (MRI) has an important complementary role to ultrasound (US) in antenatal care. Specific advantages of fetal MRI include a higher spatial resolution, excellent soft tissue contrast, the ability to acquire high‐quality images up until birth, and importantly a wide range of quantitative and functional contrasts. However, a current limitation of fetal MRI is the lack of real‐time information. While US allows for real‐time detection of, for example, reduced anatomical growth, prompting immediate additional flow assessments,^1^ MRI data is often processed offline after the patient has left the scanner, which overall limits timely clinical decision making.^2^, ^3^

This applies particularly to quantitative techniques, such as T2* relaxometry. T2* relaxometry has shown significant potential as an indirect in‐vivo evaluation of tissue oxygenation, with mean T2* values exhibiting a linear decline throughout normal gestation in the placenta^4^, ^5^, ^6^ and brain.^7^, ^8^, ^9^ In addition, reduced T2* values have been observed in pregnancies complicated by preeclampsia (PE)^10^ and FGR.^5^ Beyond the placenta and brain, T2* relaxometry has also been applied to assess the development of fetal organs, demonstrating a decrease in T2 * values throughout gestation in organs such as the lungs and thymus.^11^, ^12^

Although the acquisition of T2* data is usually fast if performed with multi‐echo gradient echo sequences (<1 min for the entire uterus at about 3 mm isotropic resolution), the subsequent steps of organ segmentation and fitting to extract quantitative information remain largely manual and time‐consuming, preventing real‐time adaptations. As a consequence, the opportunity for optimal data acquisition is lost, both regarding the addition of additional sequences as well as repetitions to mitigate artifacts (i.e., uterine contractions, reduced placental oxygenation, excessive motion), leading to sub‐optimal information available for clinical decision‐making. Furthermore, the rise of fetal interventions such as placental ablation or spina bifida repair, puts additional emphasis on real‐time information.^13^

Recent advances in artificial intelligence (AI) have demonstrated promise for overcoming these challenges. AI‐based methods have shown the ability to automatically segment placental parenchyma with high‐accuracy^14^, ^15^, ^16^ and enable subregional brain analysis.^17^ Furthermore, the feasibility of online, real‐time segmentation and quantification has been demonstrated in other fields, such as pediatric cardiac MRI^18^ and quantitative online relaxometry in adult cardiac MRI.^19^ In Fetal MRI, recent work has shown the ability to update the imaging field‐of‐view (FOV) in real‐time during gradient echo scans.^20^ Additionally, online scan planning has been demonstrated for imaging the fetal brain^21^, ^22^ and heart,^23^ paving the way for further advancements in automated and adaptive fetal imaging.

An additional development, particularly attractive for both future fetal interventions and T2* relaxometry, is the re‐emergence of lower field (0.55T) MRI. It carries a range of advantages for fetal examinations such as reduced artifacts due to increased field homogeneity, and improved patient comfort due to the larger bore size, particularly relevant for women with high body mass indices (BMI) and late gestation^24^, ^25^ and longer T2* times allowing for a larger dynamic range even in late gestation and pathological conditions.

In this work, we introduce an integrated, automated Real‐time Assessment of T2* (RAT) in the placenta and fetal brain using a 0.55T MRI scanner, producing segmented, fitted maps alongside corresponding centiles. The method was fully deployed online and evaluated prospectively in 50 fetuses (10 validation and 40 evaluation scans) over 35 weeks of gestation. The robustness of the entire real‐time pipeline was demonstrated using intra‐scan repeats, comparison with the conventional offline pipeline, and manual segmentations in the validation cohort.

## 2 METHODS

**FIGURE 1:**
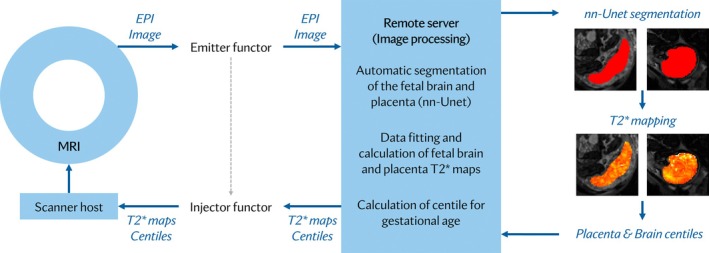
Processing pipeline for real‐time T2* assessment from the acquisition (left) to the gadgetron processing (mid) to the masks and maps (right).

The entire real‐time pipeline developed and explained in the following is graphically depicted in Figure 1.

### 2.1 Fetal Imaging and Ethics

Women were recruited to ethically approved studies (21/LO/0742 Dulwich REC, 08/12/2021; 23/LO/0685, London‐Harrow REC 14/09/2023 and 19/LO/0736, London‐Stanmore REC 20/06/2019) and informed consent was obtained. All participants were scanned in a head‐first supine position with head, back, and leg support as required. The protocol also allowed to switch to the left lateral position in cases of severe back pain or vasovagal syncope, which are commonly reported during pregnancy due to the weight of the uterus.^26^ Continuous heart rate and intermittent blood pressure monitoring were carried out in conjunction with frequent verbal interaction. A break was offered halfway through the examination.

The data sets were acquired on a commercially available 80 cm bore low‐field scanner (MAGNETOM Free.Max, Siemens Healthineers, Erlangen, Germany) using a blanket‐like 6‐element coil and a permanent 9‐element spine coil. A whole‐uterus echo planar imaging (EPI) gradient echo sequence was modified to incorporate multiple echoes and was acquired in the maternal coronal orientation. The imaging parameters included variable slices (default 62 slices), FOV =400×400 mm^2^, resolution = 3.125×3.125×3 mm^3^, Echo‐Time (TE) =[57,152,248,344] ms, Repetition Time (TR) = 2–4 s and GRAPPA=2.

### 2.2 Study Cohorts

Data sets from different cohorts, detailed in Table 1, were included in different steps of the study, for automatic deep learning segmentation of the fetal brain and placenta (training cohort), for generating retrospective control T2* normative curves (retrospective control cohort) across gestational age (GA) and to validate and evaluate the presented methodology (prospective validation cohort and prospective evaluation cohort). Medical history and outcome data were obtained from clinical records to ensure accurate classification of cases into control or non‐control categories. Cases were classified as non‐controls if they had any diagnosed brain pathology, such as ventriculomegaly or agenesis of the corpus callosum, or maternal conditions that could potentially lead to placental insufficiency, including FGR, PE, or chronic hypertension. All remaining cases, who did not meet these criteria and delivered after 36 weeks with a birth weight centile above the 3rd centile were considered controls. Cases with missing delivery information were classified as controls if no exclusion criteria were identified at the time of the scan.

**TABLE 1:** Information on Imaging contrast, Field strength, and Number of datasets included in each of the cohorts employed in this study. ssTSE: Single‐shot Turbo‐Spin Echo; bSSFP: Balanced Steady State Free Precession; MEGE: Multi‐Echo Gradient‐Echo EPI; diffusion GE: Diffusion Gradient‐Echo EPI; IRGE: Inversion Recovery Gradient‐Echo EPI.

Cohort	Imaging contrast	Field Strength	Number of datasets
Placenta Training	MEGE	0.55T, 1.5T, 3T	540
Brain Training	sSTSE, bSSFP, MEGE, diffusion GE, IRGE	0.55T, 1.5T, 3T	2989
Retrospective control	MEGE	0.55T	88
Prospective Validation	MEGE	0.55T	10
Prospective Evaluation	MEGE	0.55T	40

The retrospective control cohort consisted of 88 cases, excluding all non‐controls to ensure an accurate representation of normative T2* curves across GA. The prospective validation cohort included ten control cases, while the prospective evaluation cohort comprised 40 cases: 35 controls and 5 non‐controls diagnosed with late‐onset FGR, late‐onset PE, or hypertension.

### 2.3 Masking, T2* mapping and centile calculation

Automatic segmentation of the placenta and fetal brain was achieved using the nnU‐Net framework.^27^ For the fetal brain, a contrast‐, field strength‐, pathology‐ and age‐agnostic network was trained and tested with the above‐mentioned training cohort, composed of a total of 2989 datasets from 1045 participants, including 352, 364, and 354 ssTSE datasets, 75, 55, and 46 bSSFP datasets and 282, 314, and 420 T2* mapping datasets acquired at 0.55T, 1.5T, and 3T respectively. 120 diffusion volumes from a range of b‐values/b‐vectors and echo times and 607 T1 mapping volumes were also acquired at 0.55T.^24^, ^28^ Ground‐truth masks for this training cohort were either manually generated by a range of fetal MRI experts or automatically obtained with in‐house trained sequence‐specific validated networks. For the placenta, a multi‐field strength network was trained on a total of 540 multi‐echo gradient echo datasets from the training cohort, 180 at each field strength (0.55T, 1.5T, and 3T). Ground truth masks were generated manually by a range of fetal MRI experts using the first TE and avoiding amniotic fluid and maternal vasculature. In both networks, a split of 80%/20% for training and validation was used. All datasets in the retrospective control cohort, prospective validation, and prospective evaluation cohorts were automatically segmented with these networks. The 10 cases in the prospective validation cohort were segmented manually by an expert in fetal imaging. Dice scores between the manual segmentations and the segmentations generated by the nnU‐Net were calculated to assess the segmentation quality.

T2* fitting was performed on the multi‐echo data using least squares fitting to a monoexponential decay function. Subsequently, automatic segmentations of the fetal brain and placenta generated from the second echo of the data were applied to the T2* maps. An upper limit threshold of 500 ms for the placenta and 900 ms for the fetal brain was used to calculate the organ‐specific mean T2* values, to avoid partial volume effects from fluid‐dominated regions. On the retrospective control cohort, linear regression analysis was performed to assess the relationship between GA at scan and placental volume and mean T2* values, as well as volumes of the fetal brain, while quadratic regression analysis was applied to assess the relationship between mean T2* of the fetal brain and GA at scan. Finally, the 5th and 95th centiles were calculated for both placenta and fetal brain volume and mean T2* using quantile regression, allowing the classification into “low”, “normal”, and “high” T2*.

### 2.4 Validation

The complete real‐time implementation of the pipeline was tested on both the prospective validation and prospective evaluation cohorts. To ensure its robustness and accuracy, three different validation methods were applied to the validation cohort on top of the testing performed in the prospective evaluation cohort. First, three separate acquisitions were obtained and processed in real‐time for each case to assess the pipeline's stability across different time points (beginning, mid, and end of the scan) and its resilience to maternal or fetal movement. Second, to evaluate accuracy, automatic and manual segmentation were performed. Third, T2* mapping and centile calculations were performed both offline using the scanner‐reconstructed data and with the real‐time pipeline.

## 3 RESULTS

The described pipeline was successfully integrated into the scanner's online processing and successfully employed in all 50 participants in the prospective validation and evaluation cohorts, leading to organ‐specific T2* maps and centiles available during the scan. Table 2 provides a detailed overview of maternal characteristics and obstetric factors for both cohorts.

**TABLE 2:** Demographics including maternal characteristics, obstetric factors, and cohort type of the studied prospective cohort.

	Validation cohort (N=10)	Evaluation cohort (N=40)
**Maternal characteristics**		
Body‐mass‐index [kg/m^2^]	29.36±4.62	29.03±4.79
	[21.18−37.52]	[20.34−43.45]
Maternal age [years]	33.60±2.27	32.51±4.19
	[30−36]	[22−41]
Parity	0.10±0.32	0.09±0.28
	[0−1]	[0−1]
Gravida	1.70±0.82	1.66±1.11
	[1−3]	[1−5]
**Obstetric factors**		
Gestational age at scan	37.14±0.84	37.43±0.98
	[36−38.29]	[36−39.29]
Gestational age at delivery [weeks]	40.41±0.84	40.20±1.23
	[37.57−41.86]	[37.57−42.71]
Birthweight Centile	48.11±33.07	43.53±26.00
	[8.96−99.14]	[4.21−99.31]
APGAR score (5 min)	9.40±0.52	9.77±0.43
	[9−10]	[6−10]
**Cohort type**		
Control	*N* = 10	*N* = 35
Non‐control	*N* = 0	*N* = 5

**FIGURE 2:**
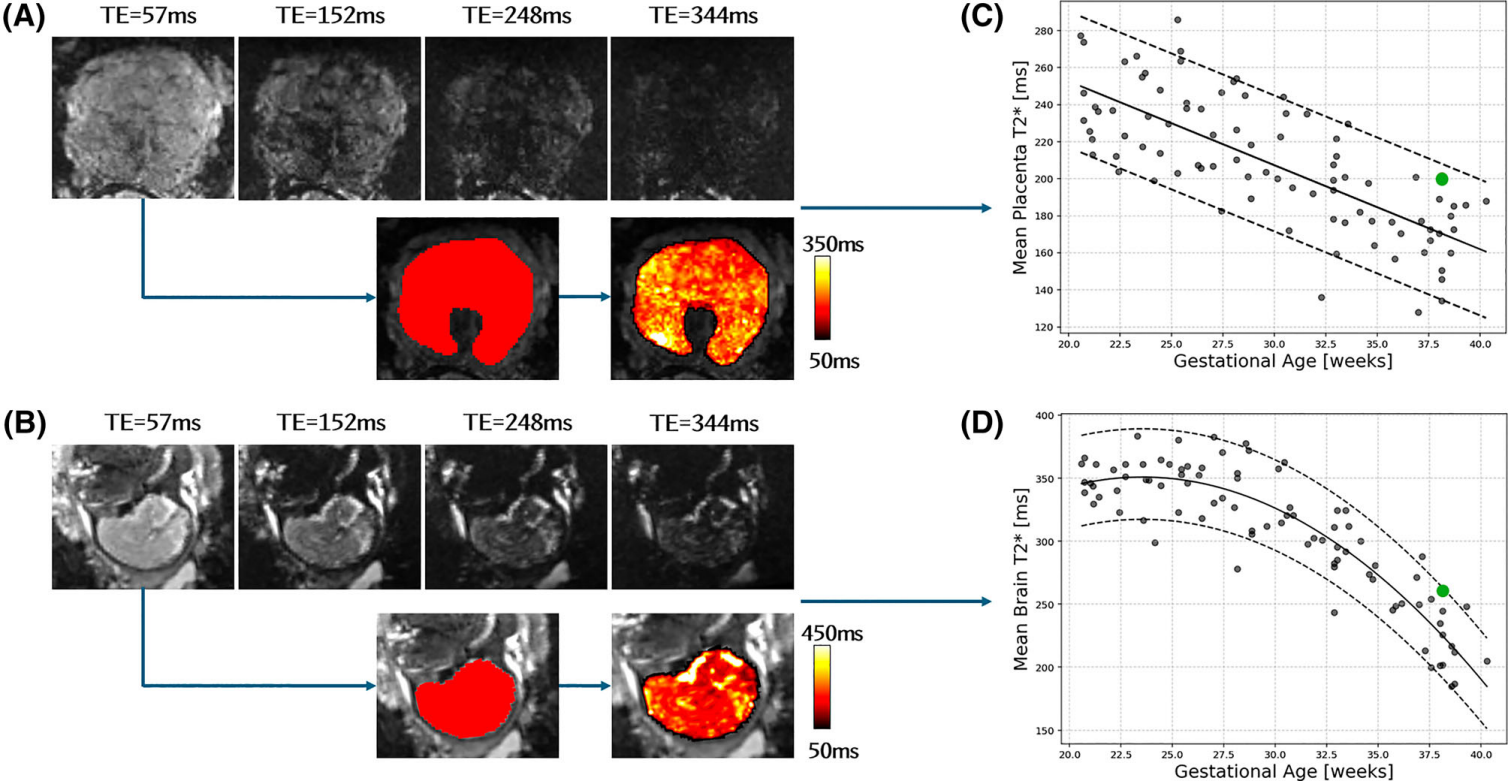
Comprehensive overview of real‐time available data for an example at 38 weeks gestation. (A and B) Coronal view of the placenta at each echo time for the placenta and fetal brain, respectively, together with organ‐specific masks in red and T2* maps in the hot color map. (C and D) Quantitative mean T2* values for the placenta and fetal brain, respectively, were plotted against the retrospective control cohort for comparison. Black dots represent the retrospective control cohort and the green dot is the specific example at 38 weeks.

Figure 2 provides a comprehensive visualization of the real‐time data obtained for an example case in the prospective validation cohort at 38 weeks GA, illustrating data acquired at multiple echo times, automatically generated masks, and T2* maps for both the fetal brain and the placenta, with quantified mean T2* values compared to normative curves from the retrospective control cohort.

**FIGURE 3:**
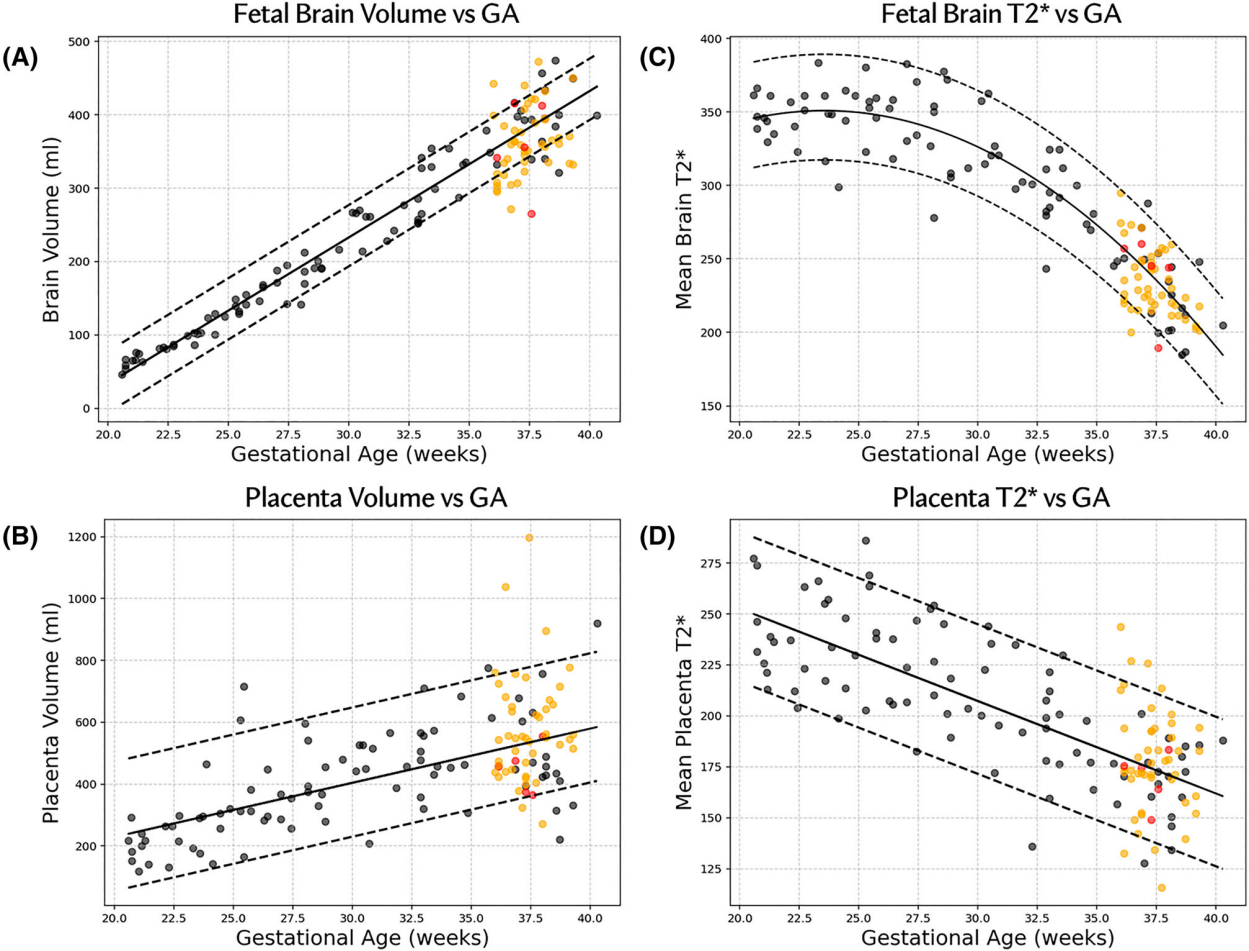
Quantitative volume and mean T2* results for the different study cohorts. All data shown for the validation and evaluation cohort were processed using automatic segmentation and quantification. (A) Fetal Brain Volume; (B) Placental Volume; (C) Fetal Brain Mean T2* and (D) Placental Mean T2*, all against gestational age (GA). Black dots represent the retrospective control cohort, orange the controls in the validation and evaluation cohorts, and red the non‐control cases in the validation and evaluation cohorts. The regression line is plotted as a black continuous line and the 5th and 95th percentiles as black dashed lines.

**FIGURE 4:**
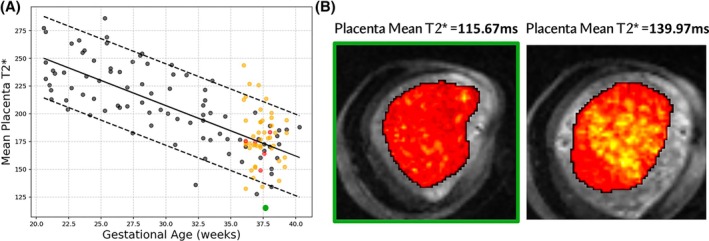
Example of a case at 37 weeks of gestation with a placental contraction during the scan. (A) Placental mean T2* during contraction depicted in green and (B) Coronal view of the placenta during contractile activity (left) and a non‐contractile state in a repeated scan (right). Black dots represent the retrospective control cohort, orange the controls in the validation and evaluation cohorts, and red the pathological cases in the validation and evaluation cohorts. The regression line is plotted as a black continuous line and the 5th and 95th percentiles as black dashed lines.

Figure 3 presents volume and mean T2* results for both the evaluation and validation cohorts, stratified into controls and non‐controls, plotted against GA, and overlaid on normative curves from the retrospective control cohort. Figure 4 presents a detailed analysis of a case from the evaluation cohort at 37 weeks of gestation, depicting a low T2* centile corresponding to variations in the uterus typically associated with contractile activity.

**FIGURE 5:**
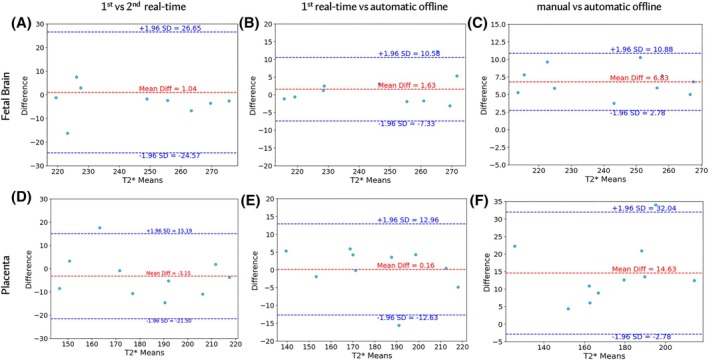
Quantitative robustness analysis using Bland‐Altman plots. (A‐D) Comparison of real‐time mean T2* values between the first and second real‐time acquisitions. (B‐E) Comparison of real‐time mean T2* values from the first real‐time acquisition with automatic offline mean T2* values from the vendor‐reconstructed acquisition. (C‐F) Comparison of manual and automatic offline mean T2* values from the vendor‐reconstructed acquisition.

The results of the robustness study are presented in Figure 5. The mean T2* difference between the first and second real‐time acquisitions is 1.04 ms for the fetal brain and −3.15 ms for the placenta. Bland‐Altman analysis comparing the mean T2* values from the first real‐time acquisition with those obtained automatically offline from the subsequent vendor‐reconstructed acquisition shows a mean difference of 1.63 ms for the fetal brain and 0.16 ms for the placenta. Additionally, the comparison of mean T2* values obtained for the offline‐reconstructed data with manual and automatic organ segmentation reveals a mean difference of 6.83 ms for the fetal brain and 14.63 ms for the placenta. The Dice score for manual and automatic segmentations was 0.84±0.02 for the placenta and 0.96±0.01 for the fetal brain.

For completeness, the robustness analysis between the intra‐scan repetitions shows similar results to the ones presented in Figure 5 with mean differences in T2* of −3.17 ms for the fetal brain and 4.74 ms for the placenta between the first and third acquisition and 5.07 ms for the fetal brain and 2.45 ms for the placenta between the second and third acquisitions. Placental centiles remained unchanged between all repeated acquisitions in all but three cases and differences are associated with visually detected contractile states of the placenta. For the fetal brain, centiles remained unchanged between repetitions in all but one case, related to increased T2* due to fetal head motion.

## 4 DISCUSSION

A fully automatic real‐time pipeline was introduced for online organ‐specific T2* quantification from a short (<30 s) whole‐uterus multi‐echo gradient echo scan. Placental and fetal brain segmentation with corresponding quantitative T2* maps and centiles were obtained in under a minute. The pipeline worked reliably in all tested cases and produced accurate segmentations in all cases for the fetal brain and all but one case for the placenta, where maternal fat had been included in the segmentation in a high BMI case. However, this issue was observed in only one of the four acquisitions, where the abdominal fat was fully within the FOV. Detecting this artifact in real‐time allowed immediate repetition. Quantitative T2* values for both brain and placenta were in‐line with previously published results on the same field strength^24^ and followed trajectories as shown in previous publications at higher field strengths.^4^, ^6^

The presented normative curves show a reduction in mean T2* for both organs over gestation in accordance with previously published work.^17^, ^29^ The higher absolute values observed in our study are due to lower field strength used as previously illustrated.^16^ The fetal brain volumes show the expected significant positive correlation with GA while the placental volumes had a general trend of increasing along with GA but with higher variability, particularly at late gestation. Differences found in T2* between the present study and our previous study published at 0.55T^28^ are likely attributable to the larger sample size, especially towards late GA. While mean fetal brain T2* follows a quite consistent trend throughout pregnancy, mean placental T2* values at late gestation (above 32 weeks) become more scattered. This could be related to the increased frequency of contractions at this GA or related to variations in placental vasculature as the time of delivery approaches. However, there is limited availability of normative placental T2* values within a large sample size to explore the reasons for this variability.

Non‐control cases in the prospective evaluation cohort were analyzed and compared to the control group. No difference was observed in the placental and brain mean T2* values for these cases, in line with the individual clinical records. In the case of late‐onset PE, clinical records indicated borderline elevated blood pressure which did not require medication for control, suggesting that the severity may not have been sufficient to affect placental function. The chronic hypertension cases were well‐managed with medication from the outset of pregnancy and had no additional complications. Additionally, one case with a prior diagnosis of FGR from an earlier US scan was included. However, both the MRI scan and a subsequent growth the US performed prior to birth showed the fetus within a normal weight centile, pointing towards a false positive result and no clinical diagnosis of FGR.

The robustness results show the consistency of the real‐time pipeline within three different multi‐echo datasets at different time points within the whole acquisition. Two cases had a difference greater than 10 ms for placental T2*, with a detailed investigation revealing that the images visually display all signs of contractile activity in individual repetitions causing a subsequent reduction in T2* as previously shown.^30^, ^31^, ^32^, ^33^ The two cases with a difference higher than 10 ms for the fetal brain were visually related to significant brain motion. The other cases showed minimal differences in mean T2* values for both the placenta and fetal brain within acquisitions with consistent centiles. The robustness shown between the presented pipeline and the offline vendor reconstructed pipeline proves the accuracy of the real‐time automatic assessment of multi‐echo data, with only two cases with a difference higher than 5 ms for the placenta due to a contraction and in the fetal brain due to motion. Centiles changed between repetitions in four cases for the placenta and one case for the fetal brain, all, however, corresponding to small mean differences and highlighting finer‐grained centile ranges as a potential future avenue. The mean Dice scores were generally high, indicating good segmentation performance, with the placenta showing a lower score due to the high variability in manual segmentations as shown before.^34^

### 4.1 Strengths and limitations

The availability of the masks, T2* maps, and centiles in real‐time during the scan is a key strength of this study, as it allows the detection of abnormal values not only due to pathologies but also secondary to contractions or fetal motion during the scan as demonstrated in the presented data. This enables an immediate reaction, for example, triggering the acquisition of extra sequences that provide additional information or the re‐acquisition of the sequence in case of contraction or motion‐corrupted images. The accurate and real‐time detection of low mean placental T2* values enables the fast detection of FGR and PE–two of the most common causes of stillbirth.^2^, ^35^, ^36^ Further promising applications could be twin‐to‐twin transfusion syndrome surgery, where previous work with fetal MRI has been undertaken offline so far.^13^, ^37^ The ability to perform this under image guidance with real‐time quantitative values might help to accurately assess the effects of the ablation in‐situ.

However, this study also has several limitations. The accuracy of placental segmentation in cases with higher BMI requires further improvement with one case showing misidentification of fat tissue. A larger number of high BMI cases is required for a comprehensive investigation. Next, the study was conducted only in late gestation due to the specific use case of late FGR. However, all networks were trained on data from the entire 2nd half of pregnancy, paving the way for future expansion. Furthermore, since only the 5th and 95th centiles were used to group the mean T2* values, some variations may be too subtle to cause a significant enough shift to detect a drop in centile. Finally, while the protocol was carefully designed to minimize noise in the images and while we have an increased dynamic range due to the reduced field strength, the noise floor was not explicitly included in the T2* fit. This could potentially lead to overestimation in severely compromised cases and late gestation cases. Future analyses will further assess this to ensure it does not introduce significant bias.

### 4.2 Implications and next steps

The next steps include work towards automatic suggestion or triggering of additional sequences in the presence of detected pathology, contraction, or motion. In addition, an increase in sample size with a higher variability will allow further assessment of pathological cohorts such as pregnancies with PE or late FGR. Moreover, including additional structural and quantitative measures such as placental location or texture may provide a more in‐depth assessment of placental function in real‐time. The method was demonstrated here for 0.55T, but was trained and developed using data from all applicable clinical field strengths to ensure broader applicability. It will be tested in the next steps on higher‐field MR scanners. Furthermore, including more high BMI cases in the placental network would enhance the robustness and generalizability of the pipeline, ensuring more reliable segmentation and T2* quantification in these cases.

## 5 CONCLUSION

The availability of quantitative organ‐specific T2* maps together with centiles based on a well‐characterized retrospective control cohort paves the way towards a more interactive fetal MRI examination, tailoring the scan for the individual, and giving the radiographers and clinicians the ability to immediately react to imaging findings and thereby provide more timely information for antenatal care. This approach is ideal for expansion to a wider usage and could enable disease‐specific biomarkers to be developed in the future.

## Conflicts of Interest

Raphael Tomi‐Tricot is an employee of Siemens Healthineers.
